# The deubiquitinase USP35: from an oncogenic hub to a therapeutic target in human cancers

**DOI:** 10.3389/fonc.2026.1825797

**Published:** 2026-05-08

**Authors:** Bisheng Li, Yuhong Wen, Rubin Wang, Jianhua Xiao, Wenjie Liao, Yihao Liao, Honggang Yuan

**Affiliations:** 1 Department of Urology, The First College of Clinical Medical Science, China Three Gorges University & Yichang Central People’s Hospital, Yichang, Hubei, China; 2 Department of Rehabilitation, Tongji Hospital, Tongji Medical College, Huazhong University of Science and Technology, Wuhan, China

**Keywords:** deubiquitinase, molecular target, tumor progression, ubiquitination, USP35

## Abstract

Ubiquitination is a critical post-translational modification in eukaryotic cells, mediated by a sequential enzymatic cascade involving E1 (activating), E2 (conjugating), and E3 (ligase) enzymes that catalyze the covalent conjugation of ubiquitin to substrate proteins. This modification precisely controls substrate stability, localization, and function, thereby governing fundamental cellular processes such as the cell cycle, DNA repair, and apoptosis. Deubiquitinating enzymes (DUBs) counteract ubiquitination by hydrolyzing ubiquitin chains, thereby maintaining cellular protein homeostasis. Ubiquitin-Specific Protease 35 (USP35), a core member of the USP family, relies on a conserved Cys-His-Asp catalytic triad and C-terminal domain (CTD)-dependent dimerization for its enzymatic activity to specifically remove ubiquitin modifications from diverse substrates. USP35 is frequently overexpressed in diverse malignancies, including clear cell renal cell carcinoma and breast cancer. By stabilizing key oncogenic factors—such as Aurora B (a proliferation regulator), NRF2 and BRD4 (inhibitors of ferroptosis), and Snail1 (a promoter of EMT)—USP35 acts as a central driver of core malignant phenotypes, including tumor cell proliferation, apoptotic evasion, metabolic reprogramming, epithelial-mesenchymal transition (EMT), and resistance to chemotherapy and immunotherapy. This review systematically summarizes the multi-dimensional regulatory mechanisms of USP35 in tumorigenesis and progression across various cancers, highlights its clinical significance as a pan-cancer prognostic biomarker and potential therapeutic target, and provides a forward-looking perspective on the development of USP35-targeted small-molecule inhibitors and combination therapies. We aim to establish a theoretical foundation and highlight translational directions for developing USP35-based precision oncology approaches.

## 1 Introduction

**Figure 1:**
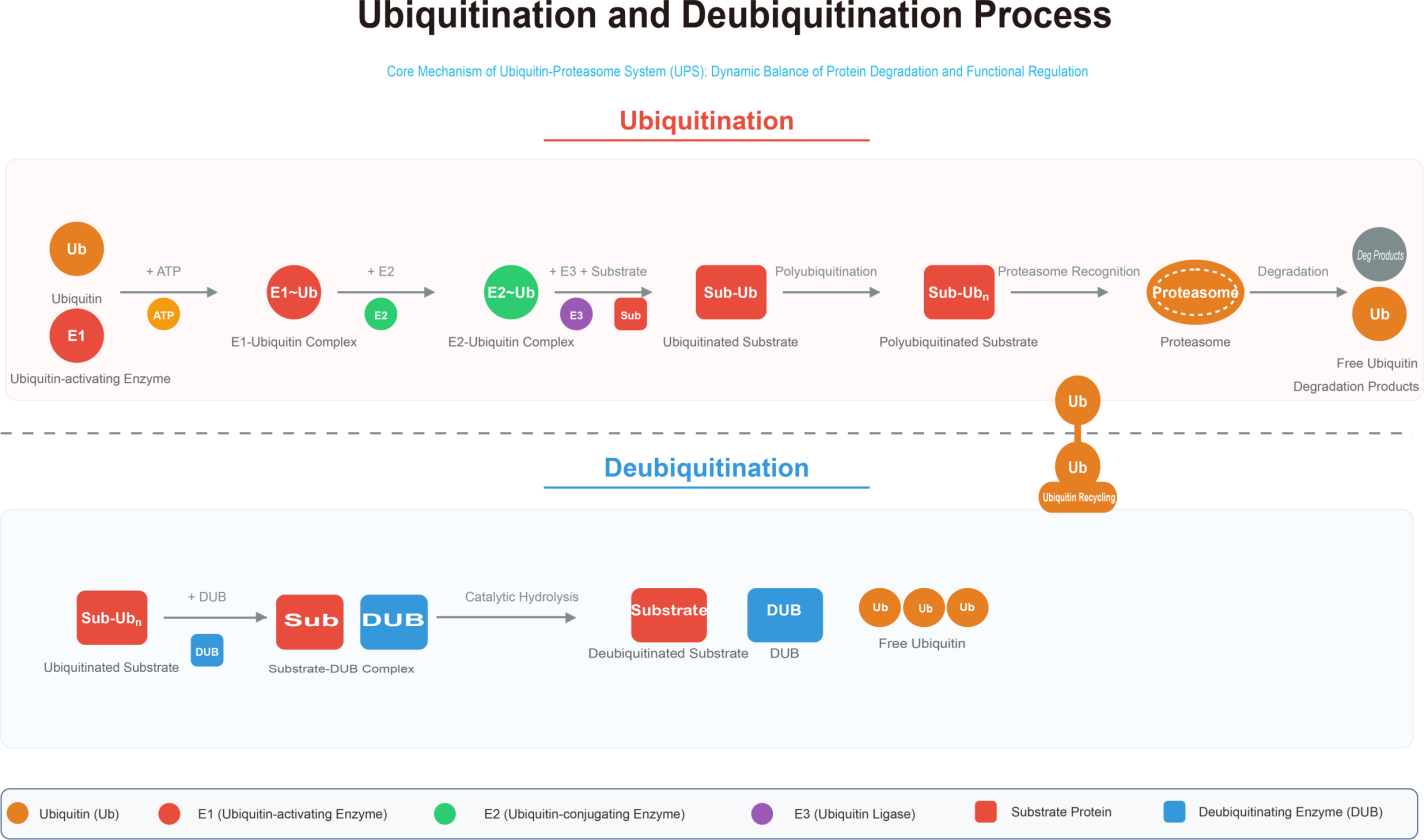
Ubiquitination and deubiquitination process. The ubiquitin-proteasome system (UPS) maintains protein homeostasis through a dynamic balance between ubiquitination and deubiquitination. The ubiquitination cascade (top) involves E1 (activating), E2 (conjugating), and E3 (ligase) enzymes that processively conjugate ubiquitin (Ub) to substrate proteins, which dictate multiple cellular outcomes: K48-linked chains primarily target substrates for proteasomal degradation, whereas other linkage types (e.g., K63) regulate non-proteolytic functions.Conversely, deubiquitinating enzymes (DUBs) catalyze the hydrolysis of ubiquitin chains (bottom), rescuing substrates from degradation and providing a critical layer of regulatory control over protein stability and function.

The precise regulation of protein function is fundamental to cellular homeostasis. Ubiquitination is a highly conserved and essential post-translational regulatory mechanism, catalyzed by a sequential cascade of ubiquitin-activating (E1), ubiquitin-conjugating (E2), and ubiquitin ligase (E3) enzymes that mediate the covalent attachment of ubiquitin to substrate proteins (1, 2). This modification governs substrate stability, subcellular localization, interactions, and activity, thereby playing a central role in diverse cellular pathways including proteasomal degradation, mitophagy, DNA damage repair, epigenetic regulation, immune responses, cell cycle progression, and apoptosis (3–6). The cellular fate of ubiquitinated substrates is precisely determined by the linkage type of the ubiquitin chain. K48-linked polyubiquitination is the canonical signal targeting substrates for proteasomal degradation, while K63-linked and other atypical ubiquitin chains mediate non-degradative outcomes such as signal transduction and protein trafficking. (**Figure 1**) Conversely, deubiquitination, catalyzed by deubiquitinating enzymes (DUBs), reverses ubiquitination by hydrolyzing isopeptide bonds within ubiquitin chains or between ubiquitin and substrates. Thus, DUBs antagonize E3 ligases, dynamically maintain protein homeostasis, and fine-tune ubiquitination-dependent signaling pathways (7, 8). categorize The human genome encodes approximately 100 DUBs, which are divided into seven structurally distinct subfamilies. Among these, the ubiquitin-specific proteases (USPs) constitute the largest and most extensively studied subgroup in cancer biology (9–11). The dynamic balance between ubiquitination and deubiquitination is crucial for cellular homeostasis (12–14), and its disruption, particularly through DUB dysfunction, is a key driver of human diseases, especially cancer, by directly promoting tumor cell proliferation, invasion, metastasis, DNA repair, metabolic reprogramming, and therapy resistance (15). Consequently, targeting DUBs has emerged as a promising therapeutic strategy for cancer intervention.

Among the various DUB subfamilies, the functional characteristics of the USP family are determined by two key domains: First, an evolutionarily conserved catalytic core, which relies on a cysteine-histidine-aspartate/asparagine (Cys-His-Asp/Asn) triad as its molecular foundation, precisely cleaves the isopeptide bond between ubiquitin and the substrate through its hydrolase activity (16–18); Second, a highly plastic regulatory domain modulates enzymatic activity strength and substrate specificity through dynamic molecular networks, including subcellular localization motifs and protein-protein interaction interfaces. By removing ubiquitin modifications from target proteins, this family regulates protein stability, thereby influencing critical cellular processes such as the cell cycle, apoptosis, DNA repair, and signal transduction, and plays a pivotal role in tumor progression (19–22). Exemplary cases include the regulation of the p53/MDM2 pathway by USP2 and USP7 (23, 24), the promotion of TGF-β signal-induced epithelial-mesenchymal transition (EMT) by USP15 and USP4 (25, 26), USP9X and USP47 regulate EMT-associated proteins such as SMAD4 and Snail, respectively (27–29), and USP28 and USP10 promote tumorigenesis through the DNA damage response and p53 pathway (30, 31).These mechanisms collectively establish the USP family as a target of significant value for cancer therapy (32–34). As a pivotal member of the USP family, USP35 has emerged as a core effector linking dysregulated ubiquitination to malignant tumor progression. Its aberrant expression and function observed across various cancer types, coupled with its ability to drive core malignant phenotypes through a complex substrate network, underpin its status as a critical regulatory interface in tumor biology and a focus for innovative targeted therapeutic strategies.

## 2 Structure and function of USP35

**Figure 2:**
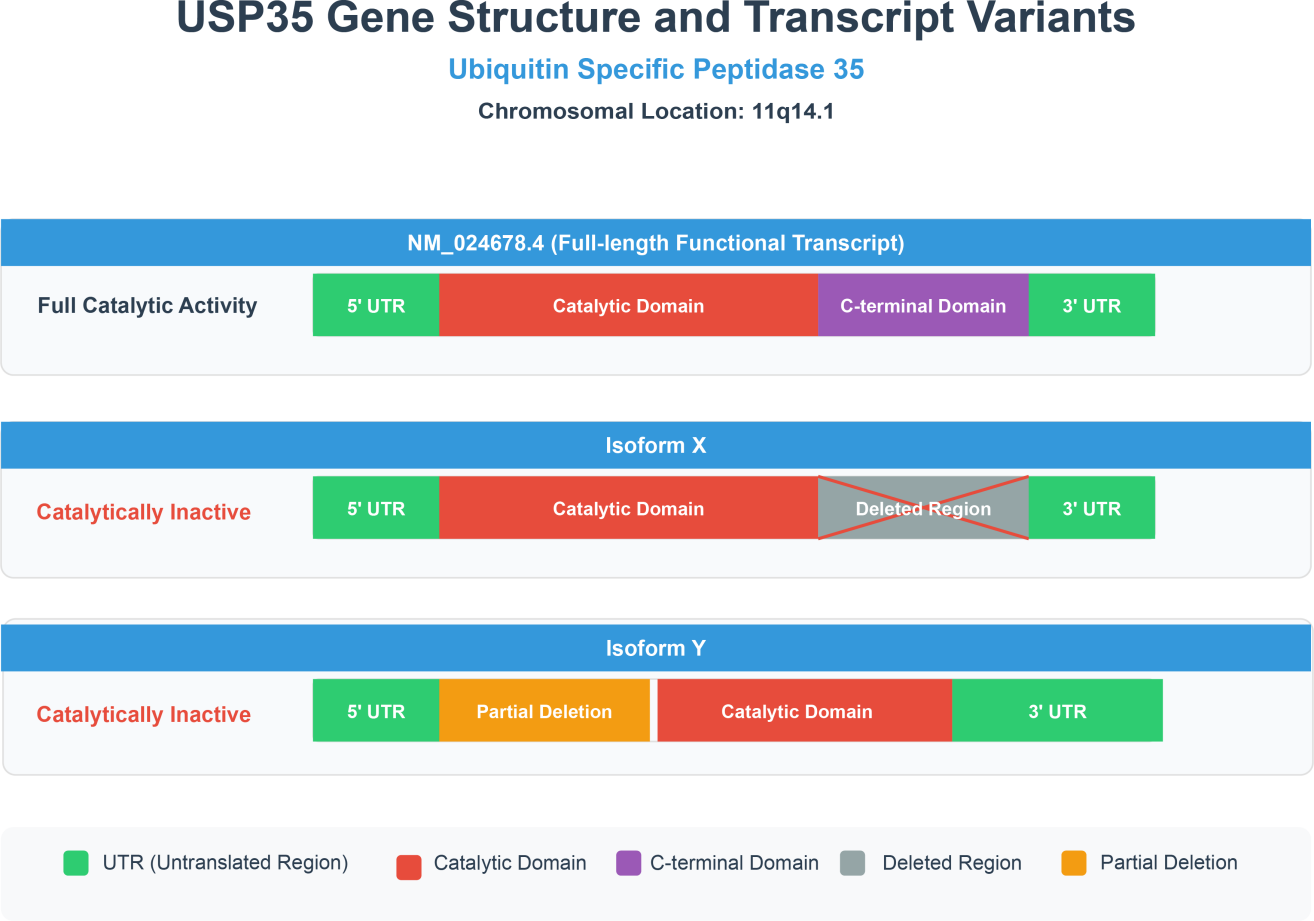
USP35 gene structure and transcript variants. Schematic representation of the USP35 gene locus (11q14.1) and its major transcript variants. The full-length functional transcript (NM_024678.4) encodes a protein with complete catalytic and C-terminal domains, essential for deubiquitination activity. In contrast, alternative splicing generates isoforms (X and Y) that harbor deletions in critical regions, resulting in the loss of catalytic function and underscoring the importance of structural integrity for USP35 enzymatic activity.

The USP35 gene is located on chromosome 11q14.1. The functional diversity of the USP35 protein arises from its complex transcriptional and post-transcriptional regulation. At least five full-length transcript variants have been identified (e.g., NM_024678.4, NM_001363763.1). The primary functional transcript encodes a 1018-amino-acid protein with a theoretical molecular mass of approximately 114 kDa. Other isoforms generated by alternative splicing often lack critical functional domains, such as parts of the C-terminal domain or insertions within the catalytic domain, and consequently, most exhibit impaired catalytic activity (35) (**Figure 2**).

The function of USP35 is dictated by the coordinated interplay of its multiple structural domains. The primary functional isoform, comprising 1018 amino acids, can be divided into four key regions:

1. N-terminal HEAT repeat domain. This region mediates subcellular localization and facilitates protein-protein interactions.
2. Core catalytic domain. This domain contains the conserved Cys-His-Asp catalytic triad (with Cys450 serving as the active-site nucleophile). Its full enzymatic activity depends on cooperative interactions with other domains.
3. Catalytic domain insertion loop. This unique structural element promotes ubiquitin binding through electrostatic interactions.
4. C-terminal domain (CTD). The CTD is essential for homodimerization, which is a prerequisite for USP35 function.

**Figure 3:**
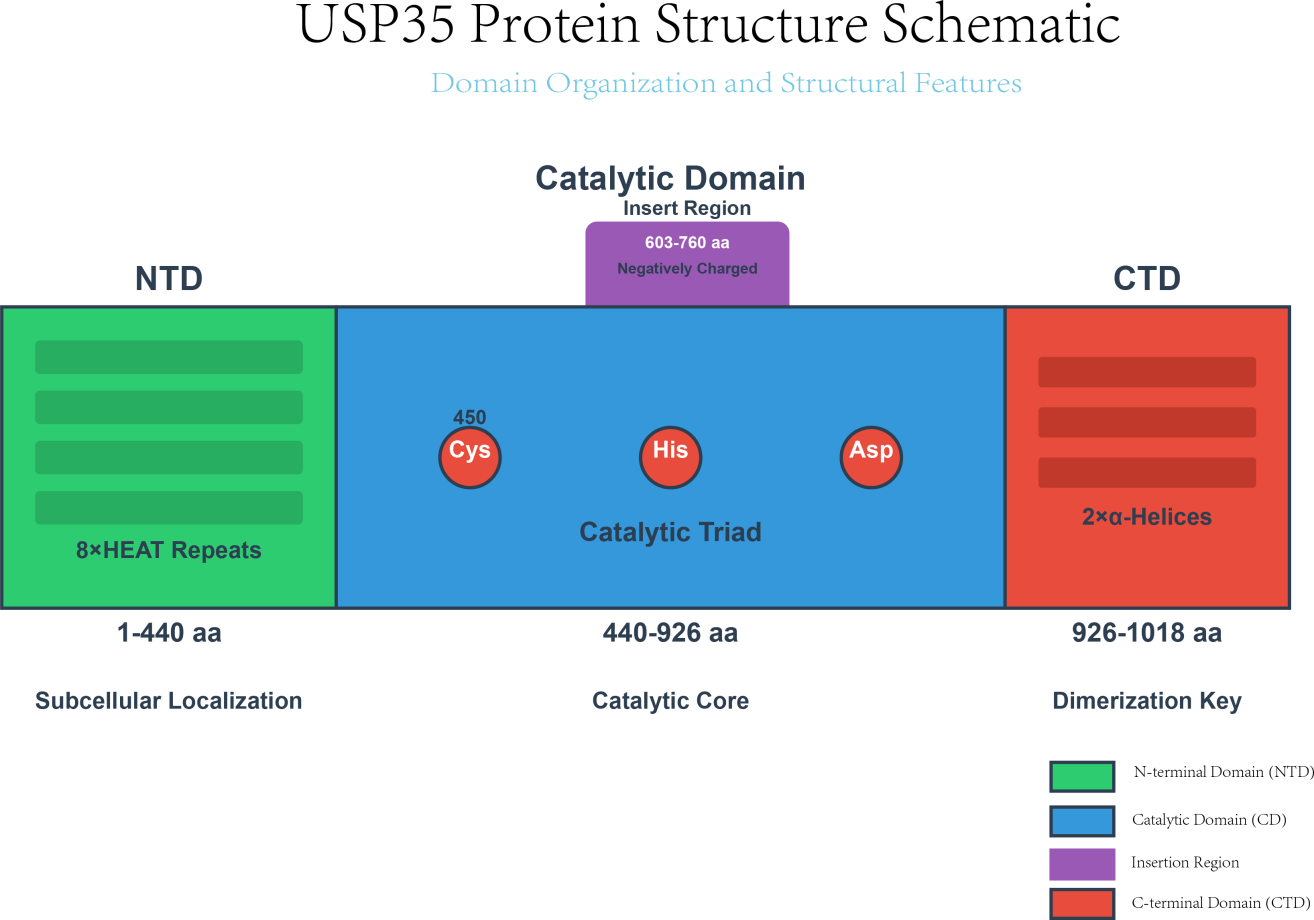
USP35 protein structure schematic. Domain organization of the functional USP35 homodimer. Key structural elements include: the N-terminal domain (NTD, aa 1-440) with HEAT repeats; the catalytic domain (CD, aa 440-926) containing the conserved Cys-His-Asp catalytic triad and a unique insertion region (aa 603-760); and the C-terminal domain (CTD, aa 926-1018). The CTD is indispensable for homodimerization, which stabilizes the protein and is a prerequisite for its full deubiquitinating enzyme activity.

From a structure-activity standpoint, CTD-mediated dimerization acts as a master structural switch. Dimer formation stabilizes an open, active conformation of the catalytic domain, ensuring the ubiquitin-binding pocket remains accessible to substrates. Conversely, deletion of the CTD disrupts dimerization, forcing the protein into a “closed” monomeric conformation. This closed state not only exhibits reduced substrate-binding affinity but also compromises the intrinsic stability of the monomer, ultimately targeting it for proteasomal degradation (35) (**Figure 3**).

USP35 regulates downstream signaling pathways by maintaining the stability of its substrate proteins. It is involved in several fundamental physiological processes: (1) Cell Cycle Regulation: USP35 stabilizes key components of the Chromosomal Passenger Complex (CPC), such as Aurora B. This stabilization maintains proper CPC localization at centromeres, thereby ensuring accurate chromosome segregation and cytokinesis and preventing G2/M phase arrest as evidenced by cell cycle defects upon USP35 depletion. (2) Mitophagy: USP35 stabilizes the mitophagy receptor FUNDC1, facilitating the clearance of damaged mitochondria via autophagosomes. This process reduces reactive oxygen species (ROS) levels and exerts a neuroprotective effect (36); Signaling Pathway Homeostasis: USP35 stabilizes ERα to modulate estrogen signaling and STING to regulate the interferon response pathway, thereby maintaining cellular signaling equilibrium (37); (4) Antiviral Response: Furthermore, USP35 may employ a similar deubiquitination mechanism to regulate other virus-associated substrates, implicating it in a broad-spectrum antiviral response (38). In tumor progression, USP35 plays a pivotal role by stabilizing a diverse array of oncoproteins, thereby driving core malignant phenotypes including uncontrolled cell proliferation, evasion of apoptosis, metabolic reprogramming, epithelial-mesenchymal transition (EMT), tumor microenvironment remodeling, and therapy resistance.

## 3 Regulation of USP35

The catalytic function of USP35 is governed not only by its multi-domain architecture but also by a sophisticated, multi-layered regulatory network operating from gene transcription to protein activity. At the transcriptional and epigenetic levels, its expression is coordinately controlled by an estrogen-ERα positive feedback loop, gene copy number amplification, and the methylation status of specific CpG sites in its promoter region (39, 40); At the post-transcriptional level, a fine-tuning layer involves microRNAs (e.g., miR-370-3p, miR-1827) which, together with USP35, form a “USP35-miRNA-MDM2” ceRNA network. Dysregulation of this network—through gene amplification or knockdown—directly disrupts cellular homeostasis, leading to consequences ranging from excessive oncoprotein stabilization to G2/M cell cycle arrest and ciliopathy-related phenotypes (16, 41); At the post-translational level, its enzymatic activity is directly enhanced by AKT-mediated phosphorylation and is also subject to indirect activation, for instance, via miR let-7a-mediated targeting of negative regulators (42); At the functional level, its substrate selectivity and catalytic efficiency depend on precise recruitment by the scaffold protein AXIN1 and subsequent liquid-liquid phase separation (LLPS) driven by TBK1-mediated phosphorylation. This process can be specifically potentiated by the small-molecule agonist KYA1797K (38). Furthermore, immune signals within the tumor microenvironment, such as IFN-γ, can indirectly upregulate USP35 expression, implicating it in immunotherapy tolerance. This multi-layered regulatory system underlies the molecular basis for the functional diversity of USP35 in determining cell fate and driving disease progression, thereby solidifying its biological status as a pivotal node across diverse cancer types.

Beyond the multi-layered regulatory mechanisms described above, USP35 itself actively shapes the TIME, thereby influencing immune surveillance and immunotherapy response. This reciprocal regulation operates at three levels. First, USP35 directly suppresses innate anti-tumor immunity.USP35 deubiquitinates and inactivates STING in ovarian cancer, blocking the STING-TBK1-IRF3 pathway and reducing type I interferon production, which leads to diminished CD8^+^ T cell infiltration (37). In melanoma, USP35 removes K63-linked ubiquitin chains from MAVS, thereby inhibiting RIG-I-MAVS signaling and suppressing IFN-β, CXCL10, and CCL5 secretion (43). Thus, tumor-intrinsic USP35 acts as a negative regulator of innate immune signaling, promoting an immunosuppressive TIME. Second, USP35 expression correlates with immune cell infiltration patterns. Pan-cancer analyses reveal that high USP35 expression is associated with reduced CD8^+^ T and NK cell infiltration, and increased M2-type tumor-associated macrophages and regulatory T cells in clear cell renal cell carcinoma. In ovarian cancer and melanoma, USP35-high tumors exhibit an immune-excluded phenotype, which is insensitive to immune checkpoint inhibitors (ICIs).Third, USP35 is a promising target to overcome ICI resistance. In melanoma models, USP35 knockout combined with oncolytic virotherapy and anti-PD−1 antibody achieves synergistic anti−tumor efficacy, converting “cold” tumors into “hot” tumors (44). In summary, USP35 not only responds to immune signals but also actively restrains anti-tumor immunity, positioning it as a rational target for combination immunotherapy.

## 4 Research progress on USP35 in various cancers

In accordance with the Reporting Recommendations for Tumor Marker Prognostic Studies (REMARK) guidelines, the independent prognostic value of USP35 across various cancer types is evaluated below based on multivariate Cox regression and ROC curve analyses where available (45).

### 4.1 Urological system

#### 4.1.1 Renal clear cell carcinoma

In clear cell renal cell carcinoma (KIRC), USP35 serves as an independent prognostic biomarker and a pivotal pro-oncogenic factor. Its expression is significantly upregulated and correlates with advanced tumor stage and poor patient prognosis. Analysis of data from The Cancer Genome Atlas (TCGA) by Guo et al. confirmed elevated USP35 expression in KIRC tissues, which positively correlates with advanced tumor stage and poor clinical outcomes. Its expression is significantly upregulated in KIRC tissues, and univariate and multivariate Cox regression analysis confirmed that high USP35 expression is an independent adverse prognostic factor for overall survival (OS) and progression-free survival (PFS) in KIRC patients (HR = 1.72, 95%CI:1.18-2.51, P = 0.005), after correcting for age, tumor stage, histological grade and other clinical covariates. ROC curve analysis further showed that USP35 has good predictive efficacy for 5-year OS of KIRC patients, with an area under the curve (AUC) of 0.731 (40). Gene Set Enrichment Analysis (GSEA) revealed that high USP35 expression enriches oncogenic pathways such as glycerophospholipid and linoleic acid metabolism, and modulates immune responses by regulating immune cell infiltration and neoantigen presentation, suggesting a role in immune evasion. Mechanistically, Wang et al. demonstrated that USP35 inhibits apoptosis by stabilizing a member of the Inhibitor of Apoptosis Proteins (IAP) family via its deubiquitinase activity (46, 47). Furthermore, USP35 deubiquitinates and stabilizes nuclear factor erythroid 2-related factor 2 (NRF2), maintaining NRF2 protein homeostasis and protecting cells from ferroptosis. Functionally, USP35 knockdown inhibits KIRC cell proliferation, migration, epithelial-mesenchymal transition (EMT), and enhances sensitivity to chemotherapeutic agents (paclitaxel, bosutinib, lapatinib) and ferroptosis inducers. *In vivo*, USP35 knockdown suppresses the growth of KIRC xenograft tumors in nude mice, underscoring its therapeutic potential (48).

#### 4.1.2 Prostate cancer (PRAD)

In prostate cancer (PRAD), USP35 is a key driver of tumor metabolic reprogramming, and its high expression correlates with poor prognosis. Multivariate Cox regression analysis, integrating age, TN stage, Gleason score and USP35 expression level, confirmed USP35 as an independent risk factor for biochemical recurrence. Time-dependent ROC curve analysis further demonstrated the predictive efficiency of USP35 in PRAD prognosis. Lin et al. demonstrated that USP35 directly deubiquitinates and stabilizes the bromodomain protein BRPF1, preventing its proteasomal degradation (49). The resulting BRPF1 accumulation drives PRAD cell proliferation, stemness, and migration/invasion. As the effector of USP35, BRPF1 binds to and activates the transcription factor SREBP2, thereby mediating the activation of the mevalonate (MVA) pathway. This USP35/BRPF1 axis sustains MVA metabolic activity in PRAD cells. Consequently, inhibition of downstream targets—either BRPF1 or the MVA pathway (e.g., using atorvastatin)—effectively suppresses the growth of USP35-high PRAD tumors (50, 51). Thus, USP35 represents a critical biomarker for MVA metabolic activity and a potential therapeutic target in PRAD.

### 4.2 Digestive system

#### 4.2.1 Esophageal cancer

In esophageal cancer, USP35 is a pivotal mediator of chemotherapy resistance by stabilizing NRF2, and its high expression predicts poor therapeutic response. Zhang et al. identified USP35 as the principal deubiquitinating enzyme regulating the stability of nuclear factor erythroid 2-related factor 2 (NRF2). Mechanistically, USP35 directly binds to NRF2 and antagonizes its ubiquitin-mediated degradation, thereby maintaining NRF2 protein levels. Functionally, USP35 knockdown reduces NRF2 expression and enhances the chemosensitivity of esophageal cancer cells (52). These findings suggest that the USP35-NRF2 axis contributes to chemotherapy resistance in esophageal cancer, and that targeting USP35 may represent a potential strategy to reverse chemoresistance in preclinical models Univariate survival analysis showed that high USP35 expression is significantly associated with poor chemotherapy response and shorter overall survival in esophageal cancer patients, while its independent prognostic value needs to be further verified by multivariate Cox regression analysis in large-scale multi-center clinical cohorts in accordance with REMARK guidelines (53).

#### 4.2.2 Gastric cancer

In gastric cancer (GC), Ma et al. identified USP35 as the principal deubiquitinating enzyme regulating the stability of Snail1, a key transcription factor governing epithelial-mesenchymal transition (EMT). Mechanistically, USP35 directly binds to Snail1 and removes its polyubiquitin chains, thereby enhancing Snail1 protein stability. Functionally, USP35 promotes gastric cancer cell invasion and migration in a deubiquitinase activity-dependent manner (54). Knockout of USP35 or depletion of Snail1 suppresses this pro-invasive and pro-migratory phenotype. *In vivo*, wild-type USP35, but not the catalytically inactive mutant (USP35-C450A), enhances the lung colonization and tumor formation of gastric cancer cells in nude mouse models. Clinically, USP35 and Snail1 expression levels are positively correlated in gastric cancer tissues, and Helicobacter pylori infection upregulates both molecules. Univariate survival analysis showed that high USP35 expression is significantly associated with lymph node metastasis, advanced TNM stage and shorter overall survival in gastric cancer patients. Its independent prognostic value needs to be further verified by multivariate Cox regression analysis with correction of age, Hp infection status, tumor stage, differentiation grade and other clinical covariates in large-scale multi-center clinical cohorts in accordance with REMARK guidelines (55). In summary, USP35 drives gastric cancer progression by deubiquitinating and stabilizing Snail1, positioning it as a potential therapeutic target.

#### 4.2.3 Hepatocellular carcinoma

High expression of USP35 in hepatocellular carcinoma (HCC) is associated with poor prognosis. Studies confirm that USP35 is upregulated in HCC tissues and cell lines, and its expression correlates with larger tumor size, advanced TNM stage, microvascular invasion, and postoperative recurrence. Prognostic assessment in the cited studies was primarily based on univariate analysis; validation of USP35 as an independent prognostic factor in HCC by multivariate Cox regression is still lacking and warrants future investigation. Mechanistically, USP35 promotes the Warburg effect (aerobic glycolysis) by deubiquitinating and stabilizing pyruvate kinase M2 (PKM2), thereby driving proliferation, migration, and invasion (56, 57). Furthermore, USP35 may enhance HCC cell proliferation by stabilizing GASC1, facilitating activation of the ROCK2 signaling pathway (58). Conversely, in Hepatitis B virus (HBV)-associated HCC, USP35 exhibits a context-dependent tumor-suppressive role. Dai et al. showed that the scaffold protein AXIN1 recruits USP35 to stabilize interferon regulatory factor 3 (IRF3) by removing K48-linked polyubiquitin chains, blocking its p62-mediated autophagic degradation. Upon HBV infection, TBK1 phosphorylates AXIN1, inducing liquid-liquid phase separation (LLPS) that enriches IRF3 and TBK1, facilitating IRF3 phosphorylation, nuclear translocation, and subsequent production of type I interferons and interferon-stimulated genes, ultimately suppressing HBV replication (38). Thus, USP35 exerts dual roles in HCC: it drives malignancy in established tumors via PKM2/GASC1, while in HBV-associated HCC, it indirectly suppresses carcinogenesis by stabilizing IRF3 to inhibit viral replication, underscoring its context-dependent functions.

#### 4.2.4 Colorectal cancer

The role of USP35 in colorectal cancer (CRC) was previously unclear. Xiao et al. confirmed that USP35 is overexpressed in CRC, promoting cell proliferation and conferring resistance to chemotherapeutic agents like Oxaliplatin (OXA) and 5-Fluorouracil (5-FU). Conversely, USP35 knockdown inhibits proliferation and enhances chemosensitivity. Using co-immunoprecipitation combined with mass spectrometry, Alpha-L-fucosidase 1 (FUCA1) was identified as a direct deubiquitination substrate of USP35. *In vitro* and *in vivo* experiments established FUCA1 as the key effector mediating USP35-driven proliferation and chemoresistance (59, 60). Furthermore, the USP35-FUCA1 axis upregulates key factors (XPC, XPA, ERCC1) in the nucleotide excision repair (NER) pathway, suggesting it confers platinum resistance by enhancing NER activity. This study elucidates that USP35 stabilizes FUCA1 to activate the NER pathway, driving CRC proliferation and chemoresistance, thereby providing a rationale for targeting this axis (61).

### 4.3 Reproductive system

#### 4.3.1 Breast cancer

USP35 plays multifaceted pro-oncogenic roles in breast cancer with subtype-specific mechanisms. In ER+ breast cancer, Cao et al. identified USP35 as a core driver through a dual mechanism: (1) USP35 stabilizes BRD4, leading to upregulated SLC7A11 expression, which consequently inhibits ferroptosis and promotes cell growth—an effect blocked by BRD4 inhibitors (39, 62). (2) USP35 and ERα engage in a positive feedback loop: USP35 stabilizes and enhances ERα transcriptional activity, while ERα signaling, together with the PI3K-AKT pathway (promoting USP35 nuclear import via AKT-mediated Ser613 phosphorylation), upregulates USP35 expression. This synergy drives rapid proliferation and confers resistance to endocrine therapies like tamoxifen (a selective estrogen receptor modulator, SERM, which competitively inhibits estrogen binding to ERα) and fulvestrant (a selective estrogen receptor degrader, SERD, which promotes ERα ubiquitination and degradation). This resistance is mechanistically linked to the USP35-mediated deubiquitination and stabilization of ERα, which sustains ERα transcriptional activity even in the presence of these antagonists. Clinical analysis confirms that high USP35 expression predicts poor prognosis (63). Across breast cancer subtypes, Lian et al. found that USP35 promotes glycolysis by modulating the ubiquitination of phosphofructokinase-1 (PFK1), supporting proliferation (64). Therapeutically, targeting USP35, especially combined with ferroptosis inducers, is promising for resistant ER+ breast cancer, particularly with PIK3CA mutations or PI3K pathway activation. Inhibiting the USP35-PFK1 axis to disrupt tumor metabolism may benefit a broader patient spectrum.

#### 4.3.2 Ovarian cancer

Ovarian cancer is the most lethal malignancy of the female reproductive system. Although the STING pathway-mediated production of type I interferons has anti-tumor activity, it is often inactivated in cancer cells (65). Zhang et al. found that USP35 is upregulated in ovarian cancer tissues, and high USP35 expression correlates with reduced CD8^+^ T cell infiltration and poor prognosis. Mechanistically, USP35 binds to and deubiquitinates STING, thereby inhibiting its activity and the subsequent STING-TBK1-IRF3 pathway activation and interferon production. Notably, STING activation promotes its phosphorylation-dependent interaction with USP35. Functionally, USP35 knockout enhances the sensitivity of ovarian cancer cells to cisplatin (37, 66, 67). Thus, USP35 upregulation constrains STING activation, positioning USP35 as a potential therapeutic target.

### 4.4 Other cancers

#### 4.4.1 Non-small cell lung cancer

USP35 is highly expressed in non-small cell lung cancer (NSCLC), where it drives malignant progression and therapy resistance by stabilizing multiple substrates. Liu et al. showed that USP35 deubiquitinates and stabilizes Vascular Endothelial Growth Factor A (VEGFA), enhancing NSCLC cell proliferation, invasion, and angiogenesis—a process positively regulated by the fusion oncoprotein FUS via maintaining USP35 mRNA stability (68). In chemotherapy resistance, USP35 stabilizes Baculoviral IAP Repeat Containing 3 (BIRC3/cIAP2) by antagonizing its K48-linked polyubiquitination, thereby inhibiting cisplatin-induced apoptosis. Clinically, USP35 and BIRC3 expression are positively correlated (69, 70). Regarding stress resistance, USP35 stabilizes Ribosome-Binding Protein 1 (RRBP1) to alleviate endoplasmic reticulum (ER) stress-induced apoptosis, and co-high expression of USP35 and RRBP1 predicts poor prognosis in lung adenocarcinoma (71, 72). Furthermore, USP35 inhibits ferroptosis by stabilizing ferroportin (FPN), promoting cancer cell survival upon induction (e.g., with erastin or RSL3) and tumor growth. Notably, USP35 knockdown sensitizes NSCLC cells to cisplatin and paclitaxel (73). Thus, through its multi-target actions—promoting tumorigenesis (VEGFA), conferring cisplatin resistance (BIRC3), countering ER stress (RRBP1), and suppressing ferroptosis (FPN)—USP35 constitutes a core regulatory network in NSCLC, making it a promising therapeutic target. The recent identification of chidamide as a functional USP35 antagonist further validates its therapeutic potential, as will be discussed in Section 6 (74).

#### 4.4.2 Melanoma

In cutaneous melanoma (SKCM), the RIG-I–MAVS antiviral signaling pathway is regulated by ubiquitination. While E3 ligases like TRIM31 and TRIM21 activate MAVS, the deubiquitinase USP35 negatively regulates the pathway by removing K63-linked ubiquitin chains from MAVS, thereby suppressing MAVS activation and subsequent interferon signaling (75, 76). Zhang et al. further demonstrated that USP35 overexpression in SKCM is associated with poor prognosis, mTORC1 pathway activation, and tumor microenvironment remodeling. High USP35 expression correlates with reduced CD8^+^ T cell infiltration and diminished response to immunotherapy (58). Conversely, in animal models, USP35 knockout combined with oncolytic virotherapy enhances CD8^+^ T cell infiltration and the secretion of IFN-β, CXCL10, and CCL5, suppressing tumor progression (43). Therefore, targeting USP35 to restore MAVS activity and enhance CD8^+^ T cell infiltration represents a promising strategy to overcome immunotherapy resistance in melanoma.

#### 4.4.3 Lymphoma

**Figure 4:**
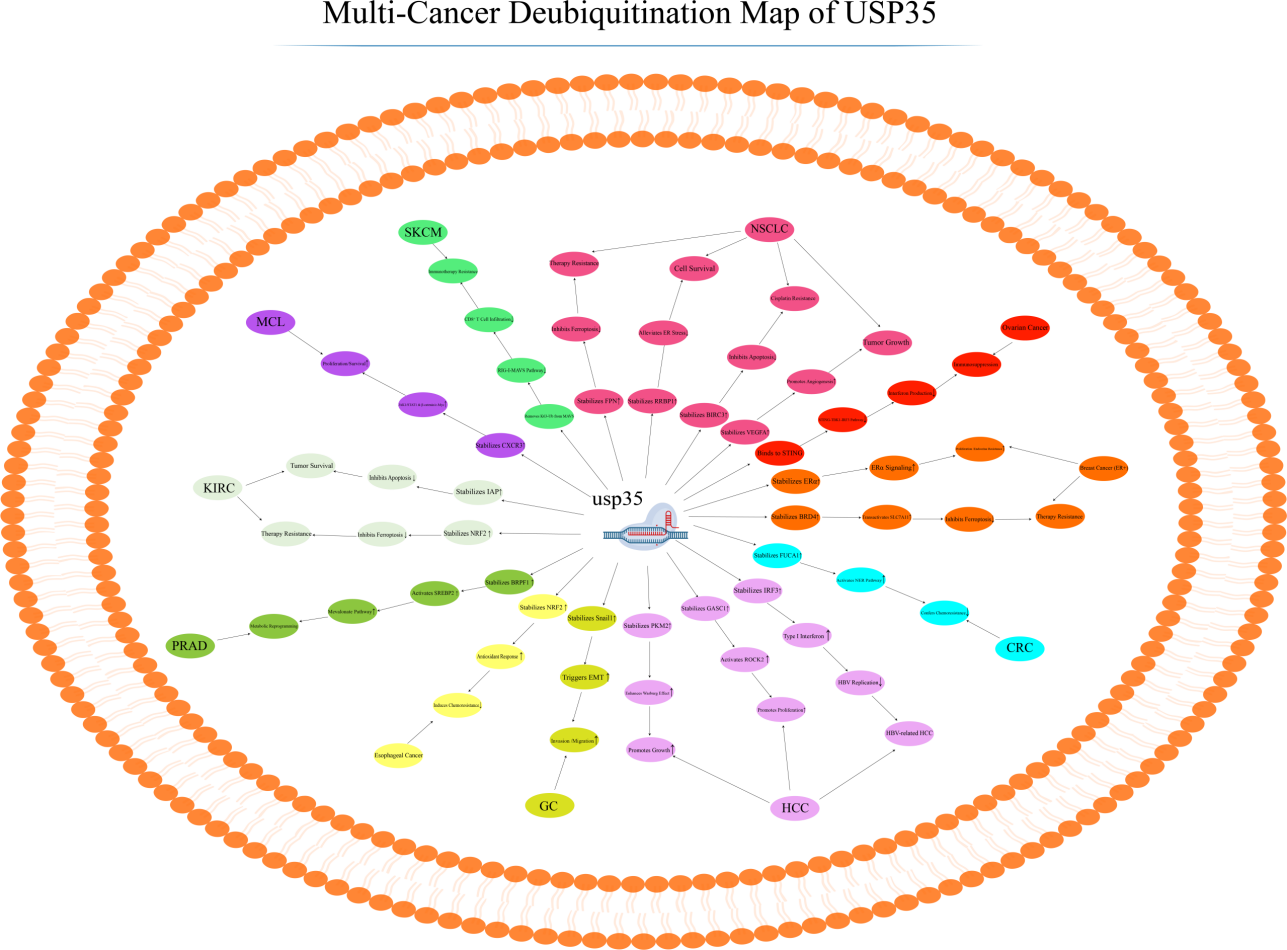
Pleiotropic regulatory mechanisms of USP35 in tumors. USP35 drives multiple hallmarks of cancer through the deubiquitination and stabilization of specific oncoproteins across diverse tumor types. This coordinated action enhances tumor cell proliferation, metabolic reprogramming, epithelial-mesenchymal transition (EMT), resistance to cell death and therapy, and remodeling of the tumor microenvironment, thereby positioning USP35 as a central node in oncogenesis and a promising therapeutic target.

Mantle cell lymphoma (MCL) is an aggressive B-cell non-Hodgkin lymphoma with a poor prognosis, and treatment options are limited by resistance and relapse (77, 78). Zou et al. discovered that USP35 directly binds to and deubiquitinates CXCR3 in MCL, stabilizing its expression by blocking ubiquitin-proteasome-mediated degradation. Functionally, the USP35-CXCR3 axis activates JAK1/STAT1 signaling and upregulates β-catenin and c-Myc, promoting tumor cell proliferation, migration, and invasion while inhibiting apoptosis. CXCR3 is overexpressed in MCL, and its gene amplification is associated with blastoid/pleomorphic variants. Experimentally, USP35 knockdown reduces CXCR3 levels and suppresses tumor growth, whereas CXCR3 overexpression rescues the effects of USP35 loss. Thus, the USP35-CXCR3 axis is critical in MCL progression, offering a potential target for patients with high CXCR3 expression or amplification (79) (**Figure 4**) (**Table 1**).

**Table 1:** Research progress of USP35 in different cancers.

Tumor system	Tumor type	Key mechanisms of USP35	Key factors	Effect	References
Urinary System	Clear Cell Renal Cell Carcinoma	• Deubiquitinates and stabilizes IAP family members, inhibiting apoptosis • Deubiquitinates and stabilizes NRF2, counteracting ferroptosis • Enriches glycerophospholipid/linoleic acid metabolic pathways, regulating immune evasion	IAP, NRF2, glycerophospholipid metabolism-related proteins, linoleic acid metabolism-related proteins	↑ (Oncogenic)	(40, 46–48)
Urinary System	Prostate Cancer	Deubiquitinates and stabilizes BRPF1, activating SREBP2 to mediate mevalonate (MVA) metabolic pathway activation	BRPF1, SREBP2, MVA pathway-related proteins	↑ (Oncogenic)	(49–51)
Digestive System	Esophageal Cancer	Deubiquitinates and stabilizes NRF2, antagonizing its ubiquitin-mediated degradation and maintaining NRF2 protein levels	NRF2	↑ (Oncogenic)	(52, 53)
Digestive System	Gastric Cancer	Deubiquitinates and stabilizes the EMT transcription factor Snail1, enhancing its protein stability (Helicobacter pylori upregulates both expressions)	Snail1	↑ (Oncogenic)	(54, 55)
Digestive System	Hepatocellular Carcinoma - Oncogenic	• Deubiquitinates and stabilizes PKM2, activating the Warburg effect • Deubiquitinates and stabilizes GASC1, activating the ROCK2 pathway	PKM2, GASC1, ROCK2	↑ (Oncogenic)	(56–58)
Digestive System	Hepatocellular Carcinoma - Indirect Tumor-Suppressive	Forms complex with AXIN1, deubiquitinates and stabilizes IRF3 (blocking p62-mediated degradation), activating type I interferon pathway and inhibiting HBV replication	AXIN1, IRF3, TBK1, IFN-β, IFN-α4, ISG15, CXCL10	↓ (Tumor-Suppressive)	(42)
Digestive System	Colorectal Cancer	Deubiquitinates and stabilizes FUCA1, upregulating NER pathway (XPC/XPA/ERCC1) and enhancing platinum resistance	FUCA1, XPC, XPA, ERCC1	↑ (Oncogenic)	(59–61)
Reproductive System	Breast Cancer - ER+ subtype	• Deubiquitinates and stabilizes BRD4, upregulating SLC7A11 and inhibiting ferroptosis • Forms positive feedback loop with ERα (AKT phosphorylates USP35 at Ser613), mediating endocrine resistance	BRD4, SLC7A11, ERα, AKT (phosphorylates USP35 at Ser613)	↑ (Oncogenic)	(39, 62, 63)
Reproductive System	Breast Cancer - Pan-subtype	Deubiquitinates and stabilizes PFK1, promoting glycolysis and supporting cancer cell proliferation	PFK1	↑ (Oncogenic)	(64)
Reproductive System	Ovarian Cancer	Deubiquitinates and inhibits STING activity, blocking STING-TBK1-IRF3 pathway and reducing type I interferon production	STING, TBK1, IRF3	↑ (Oncogenic)	(37, 65–67)
Other Tumors	Non-Small Cell Lung Cancer	• Deubiquitinates and stabilizes VEGFA (FUS regulates USP35 mRNA), promoting angiogenesis • Deubiquitinates and stabilizes BIRC3, antagonizing cisplatin-induced apoptosis • Deubiquitinates and stabilizes RRBP1, resisting ER stress-induced apoptosis • Deubiquitinates and stabilizes FPN, inhibiting ferroptosis	VEGFA, FUS, BIRC3, RRBP1, FPN	↑ (Oncogenic)	(68–73)
Other Tumors	Melanoma	Deubiquitinates and removes K63-linked ubiquitin chains from MAVS, inhibiting RIG-I-MAVS pathway and reducing type I interferon secretion	MAVS, RIG-I, TBK1, IRF3/IRF7, IFN-β, CXCL10, CCL5	↑ (Oncogenic)	(43, 58, 74–76)
Other Tumors	Mantle Cell Lymphoma	Deubiquitinates and stabilizes CXCR3, activating JAK1/STAT1 pathway and upregulating β-catenin and c-Myc	CXCR3, JAK1, STAT1, β-catenin, c-Myc	↑ (Oncogenic)	(77–79)

Color Legend: Urinary System, Digestive System, Reproductive System, Other Tumors, Oncogenic Effect (↑), Tumor-Suppressive Effect (↓).

A systematic summary of USP35’s oncogenic roles across various tumor systems. The table delineates how USP35 stabilizes distinct substrate proteins to activate specific pathways, thereby promoting core cancer hallmarks such as proliferation, apoptosis evasion, and chemoresistance. A notable context-dependent, tumor-suppressive function is observed in HBV-associated hepatocellular carcinoma, where USP35 stabilizes IRF3 to enhance antiviral innate immunity. “↑” = oncogenic effect, “↓” = tumor-suppressive effect.

## 5 Other aspects

**Figure 5:**
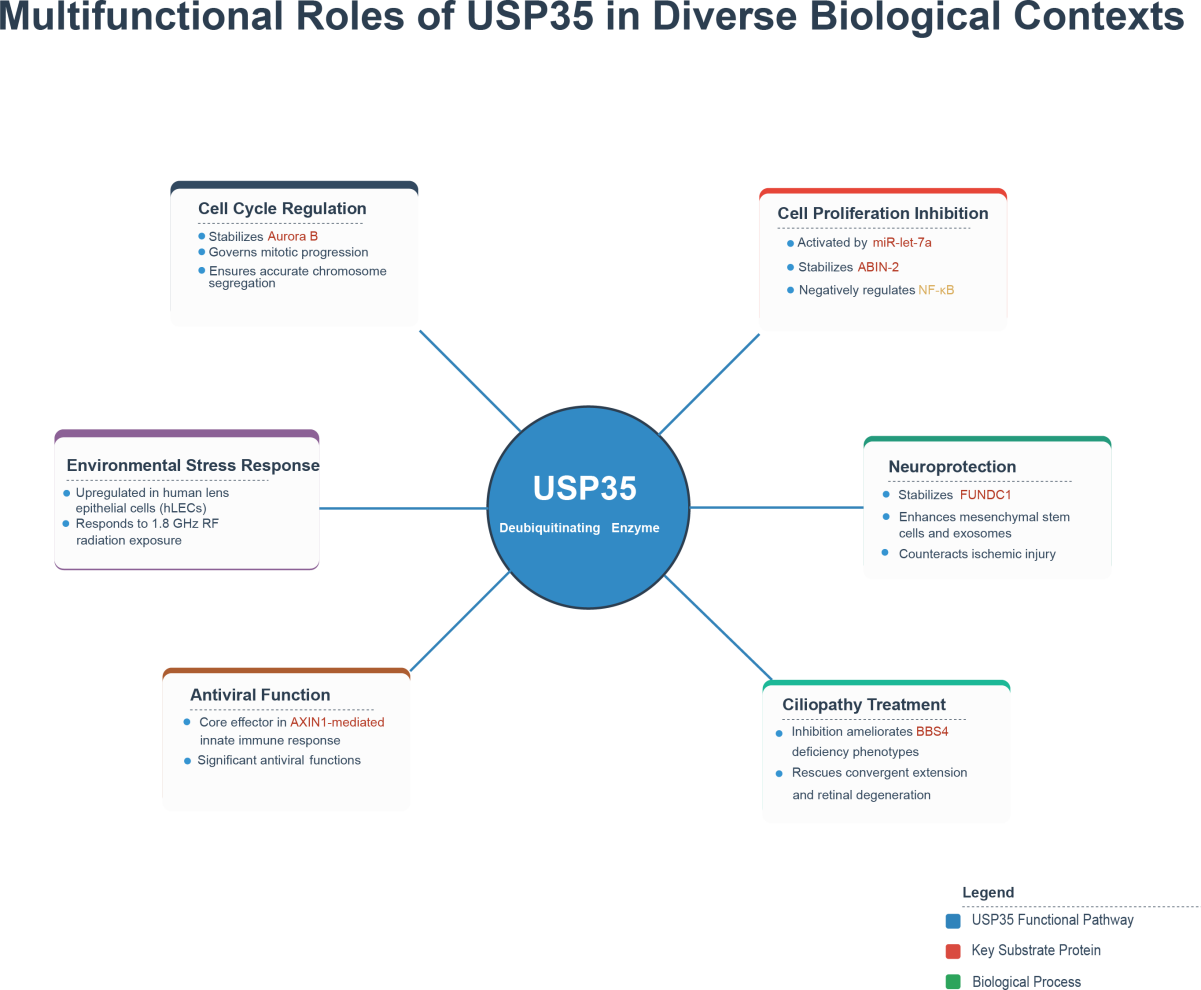
Multifunctional roles of USP35 in diverse biological contexts. USP35 exerts pleiotropic biological functions by stabilizing distinct substrate proteins in specific cellular contexts: it ensures mitotic fidelity via Aurora B, modulates inflammatory signaling through ABIN-2/NF-κB axis, promotes mitochondrial quality control and neuroprotection by stabilizing FUNDC1, facilitates antiviral innate immunity within the AXIN1-mediated pathway, maintains cellular homeostasis under environmental stress, and rescues ciliopathy-related defects, highlighting its role as a context-dependent regulatory hub in cellular physiology.

Ubiquitin Specific Peptidase 35 (USP35), as a member of the deubiquitinating enzyme family, has demonstrated functional diversity across multiple biological contexts, underscoring its potential as a pivotal regulatory node. (**Figure 5**) At the fundamental cell biology level, USP35 influences core cellular processes through the precise regulation of key substrate stability: it governs mitotic progression by stabilizing the mitotic kinase Aurora B (80);Upon activation by miR-let-7a, USP35 subsequently stabilizes the ABIN-2 protein, thereby inhibiting cell proliferation and negatively regulating the activation of the ID signaling pathway (81). In the context of neuroprotection, USP35 stabilizes FUNDC1 through deubiquitination, thereby enhancing the capacity of mesenchymal stem cells and their exosomes to counteract ischemic-reperfusion-induced neuronal injury (36). Furthermore, inhibition of USP35 function can ameliorate ciliopathy-related phenotypes caused by BBS4 deficiency, such as convergent extension defects and retinal degeneration, highlighting its critical role in this class of diseases (41). In the field of virus-host interactions, USP35 demonstrates significant antiviral functions, serving as a core effector in the AXIN1-mediated innate immune response against viruses (38). Furthermore, USP35 exhibits responsiveness to environmental stress. Studies have confirmed it to be one of the significantly upregulated protein biomarkers in human lens epithelial cells (hLECs) following exposure to 1.8 GHz radiofrequency radiation at specific absorption rates of 3 and 4 W/kg for 2 hours (82). In summary, current research consistently demonstrates that the core mechanism of USP35 lies in its dynamic regulation of the abundance and function of key effector proteins via its deubiquitinase activity, thereby enabling its broad involvement in cell cycle control, proliferation inhibition, antiviral defense, and stress responses. It is particularly noteworthy that its regulatory roles in processes such as cell proliferation (e.g., via ABIN-2) and critical survival/inflammatory signaling pathways (e.g., NF-κB) are highly relevant to the core mechanisms of tumorigenesis and progression. Therefore, systematically elucidating the precise functions of USP35 in tumor biology, its regulatory networks, and its potential as a therapeutic target represents a research direction of significant current value.

## 6 USP35-targeted therapeutic strategies from bench to bedside

The compelling evidence positioning USP35 as a central oncogenic driver across numerous cancers has naturally sparked intense interest in its therapeutic potential. Translating this wealth of biological knowledge into clinical applications is the next critical frontier. Current efforts are focused on three progressive avenues: the identification of existing small molecules with anti-USP35 activity, the development of advanced drug platforms for its selective degradation, and the design of next-generation inhibitors based on its unique structural biology.

### 6.1 Repurposing and *De Novo* discovery of USP35 inhibitors

The path to targeting USP35 is being paved through both serendipitous drug repurposing and dedicated discovery campaigns. A landmark study has identified that the clinically approved histone deacetylase inhibitor (HDACi), Chidamide, functions as a direct USP35 antagonist. In lung cancer models, Chidamide was shown to physically bind to USP35 and reduce its protein expression. This interaction is functionally significant: by downregulating USP35, Chidamide effectively impedes glycolysis, promotes ferroptosis, and, most importantly, markedly enhances the sensitivity of lung cancer cells to cisplatin (DDP) (74). *In vivo* xenograft models further corroborated that Chidamide treatment reduced tumor growth and potentiated the efficacy of DDP. This discovery serves as a powerful proof-of-concept that USP35 is a “druggable” target and highlights an immediate translational pathway for repurposing Chidamide in USP35-overexpressing malignancies.

### 6.2 PROTACs and nanocarrier-enabled delivery

Proteolysis-Targeting Chimeras (PROTACs) offer a revolutionary therapeutic modality that surpasses the limitations of traditional occupancy-driven inhibitors (83, 84). Instead of merely blocking USP35’s enzymatic activity, PROTACs are heterobifunctional molecules designed to recruit an E3 ubiquitin ligase to USP35, inducing its proximity-dependent polyubiquitination and subsequent proteasomal degradation. This event-driven, catalytic mechanism leads to the complete and sustained elimination of the target protein, potentially ablating all its oncogenic functions—both catalytic and scaffolding (85).

The feasibility of this approach for the DUB family is well-established. Pioneering work on USP7 has demonstrated that highly selective PROTAC degraders can be developed, which successfully induce apoptosis in USP7-dependent cancer cells (86). These studies validate the core principle of DUB-targeted degradation and provide a clear methodological blueprint for developing analogous USP35 degraders. Furthermore, recent reviews underscore the immense potential of targeted protein degraders, including PROTACs and molecular glues, as next-generation strategies for drugging USPs across various cancers (87). Applying this platform to USP35 could yield highly potent and selective agents, overcoming potential resistance mechanisms associated with enzymatic inhibition.

Beyond PROTAC design, clinical translation of USP35-targeted degraders will require robust delivery platforms. PROTACs remain constrained by high molecular weight, limited aqueous solubility, and suboptimal tumor biodistribution (88). Nanomedicine offers a viable solution: lipid-based, polymeric, and inorganic nanocarriers can encapsulate PROTACs to enhance systemic stability, enable tumor-specific accumulation via the enhanced permeability and retention (EPR) effect, and achieve spatiotemporally controlled release in response to tumor microenvironmental stimuli (89, 90). Furthermore, recombinant bioPROTACs delivered by biocompatible lipid nanoparticles achieve near-complete elimination of target proteins within hours of treatment (91). Notably, engineered nanoparticles can intrinsically reshape the immunosuppressive tumor microenvironment, converting immunologically “cold” tumors into “hot” ones that respond more effectively to immunotherapy (92, 93). Integrating these delivery innovations with USP35-targeted degraders may accelerate bench-to-bedside translation by overcoming the pharmacological barriers that currently impede clinical application of DUB-directed therapies.

### 6.3 Exploiting the dimerization interface for next-generation inhibitors

The strict dependence of USP35 enzymatic activity on C-terminal domain (CTD)-mediated homodimerization presents a structural vulnerability amenable to allosteric targeting. Disruption of this dimer interface offers a route to selective inhibition that circumvents the off-target toxicity associated with conserved catalytic pockets—a challenge underscored by the clinical failure of the broad-spectrum DUB inhibitor VLX1570 due to pulmonary toxicity (94). Beyond inhibition, pharmacological modulation of USP35 activity may also hold therapeutic value, as illustrated by the small-molecule agonist KYA1797K, which potentiates the AXIN1-USP35-IRF3 axis via enhanced liquid-liquid phase separation (38). Although preclinical, this agonist strategy suggests that context-dependent activation of USP35 could be exploited in antiviral immunity or rationally designed combination regimens.

## 7 Conclusions, challenges, and future directions

Ubiquitination homeostasis is fundamental to cellular integrity, and its dysregulation is a recognized hallmark of cancer. This review synthesizes compelling evidence that positions the deubiquitinase USP35 as a pivotal oncogenic driver and a promising therapeutic target across diverse malignancies. Its frequent overexpression and correlation with poor clinical outcomes underscore its potential as a pan-cancer biomarker. To fully realize this therapeutic promise, future research must address critical challenges across basic and translational science.

### 7.1 Deepening mechanistic understanding to navigate the complex tumor landscape

A deeper, more nuanced understanding of USP35 biology within the complex tumor ecosystem is paramount. This will be driven by the strategic application of cutting-edge technologies.

Harnessing Multi-Omics and Single-Cell Technologies: Bulk sequencing data, such as from TCGA, has established USP35 as a pan-cancer gene associated with poor prognosis in cohorts like skin cutaneous melanoma (SKCM). However, to dissect its role in tumor heterogeneity, future studies must leverage single-cell RNA sequencing (scRNA-seq). This technology can precisely map USP35 expression across diverse cell populations within the TME, such as malignant cells, immune subsets (e.g., CD8+ T cells, Tregs, TAMs), and stromal cells (95). Complementing this, spatial transcriptomics will add a crucial layer of geographical context, revealing how USP35 expression in tumor cells influences neighboring immune and stromal architecture.

Deciphering the Immunosuppressive Network: Emerging evidence positions USP35 as a key orchestrator of an immunosuppressive TME. In SKCM, high USP35 expression is significantly associated with a negative correlation in CD8+ T cell infiltration and a dampened response to immunotherapy. Mechanistically, USP35 achieves this by deubiquitinating and stabilizing inhibitor of DNA binding 3(ID3), which in turn upregulates PD-L1 expression to facilitate immune escape. This is further validated by the discovery that the USP35 inhibitor IU1 can suppress ID3 expression, reduce PD-L1 levels, and augment the efficacy of PD-L1 monoclonal antibody therapy (96). Systematic investigation into how USP35 regulates other immune checkpoints and cytokine networks will be crucial for developing effective immunotherapy combinations.

### 7.2 Overcoming translational hurdles from bench to bedside

Translating preclinical findings into clinical utility requires systematic validation. First, establishing USP35 as an independent prognostic biomarker demands large-scale, multi-center cohort studies employing standardized assays and multivariate Cox regression adjusted for clinicopathological covariates, in accordance with REMARK guidelines. Parallel development of a validated companion diagnostic is essential for patient stratification. Clinical evaluation of USP35-targeted agents should proceed through phase I/II trials to define safety, dosing, and preliminary efficacy in molecularly defined populations, with definitive evidence emerging from randomized controlled trials comparing USP35-directed combinations against standard-of-care regimens using robust endpoints such as progression-free and overall survival. This evidence-based framework is critical to advancing USP35 from a compelling target to a clinically actionable therapeutic strategy.

### 7.3 Rational combination strategies for the future of precision medicine

The ultimate goal is to translate USP35’s biology into personalized cancer care through the rational design of combination therapies tailored to specific tumor contexts. Based on the current evidence, combining USP35 inhibition with standard-of-care chemotherapeutics such as cisplatin represents a promising approach to reverse USP35-mediated drug resistance, as demonstrated in lung cancer models. In parallel, co-administration of USP35 inhibitors with anti-PD-1/PD-L1 immune checkpoint blockade may reshape the immunosuppressive tumor microenvironment, converting immunologically “cold” tumors into “hot” ones and thereby unleashing durable anti-tumor immunity, a strategy supported by findings in cutaneous melanoma and colorectal cancer. Furthermore, exploring synergistic combinations with agents that target downstream effectors or parallel oncogenic networks could maximize therapeutic impact while forestalling the emergence of acquired resistance.

In conclusion, this review establishes a cohesive “structure-function-disease” paradigm for USP35. By addressing the specific technological and translational priorities outlined herein, the field is poised to transform USP35 from a compelling molecular target into a clinically actionable strategy that may guide both prognostic assessment and the rational deployment of targeted combination therapies.

## Glossary

APC/C: Anaphase-Promoting Complex/Cyclosome
AUC: Area Under the Curve
BIRC3: Baculoviral IAP Repeat Containing 3
BRD4: Bromodomain-containing protein 4
ceRNA: Competing endogenous RNA
CPC: Chromosomal Passenger Complex
CRC: Colorectal Cancer
CTD: C-terminal domain
DUB: Deubiquitinating enzyme
EMT: Epithelial-Mesenchymal Transition
ER: Estrogen Receptor
FPN: Ferroportin
FUCA1: Alpha-L-fucosidase 1
HBV: Hepatitis B virus
HCC: Hepatocellular Carcinoma
HDACi: Histone deacetylase inhibitor
HR: Hazard Ratio
IAP: Inhibitor of Apoptosis Proteins
ICI: Immune Checkpoint Inhibitor
ID3: inhibitor of DNA binding 3
IFN: Interferon
IHC: Immunohistochemistry
IRF3: Interferon Regulatory Factor 3
KIRC: Kidney Renal Clear Cell Carcinoma
LLPS: Liquid-Liquid Phase Separation
MAVS: Mitochondrial Antiviral-Signaling Protein
MCL: Mantle Cell Lymphoma
MVA: Mevalonate
NER: Nucleotide Excision Repair
NRF2: Nuclear factor erythroid 2-related factor 2
NSCLC: Non-Small Cell Lung Cancer
OS: Overall Survival
PD-1/PD-L1: Programmed cell death protein 1/Programmed death-ligand 1
PFS: Progression-Free Survival
PKM2: Pyruvate kinase M2
PRAD: Prostate Adenocarcinoma
PROTAC: Proteolysis-Targeting Chimera
REMARK: Reporting Recommendations for Tumor Marker Prognostic Studies
RIG-I: Retinoic acid-inducible gene I
ROC: Receiver Operating Characteristic
ROS: Reactive Oxygen Species
scRNA-seq: Single-cell RNA sequencing
SERD: Selective Estrogen Receptor Degrader
SERM: Selective Estrogen Receptor Modulator
SKCM: Skin Cutaneous Melanoma
STING: Stimulator of Interferon Genes
TCGA: The Cancer Genome Atlas
TIME: Tumor Immune Microenvironment
TNM: Tumor, Node, Metastasis
UPS: Ubiquitin-Proteasome System
USP: Ubiquitin-Specific Protease
VEGFA: Vascular Endothelial Growth Factor A

## References

[1] Keijzer N Sakoltchik J Majumder K van Lil N El Oualid F Fish A . Usp1/Uaf1 targets polyubiquitinated Pcna with an exo-cleavage mechanism that can temporarily enrich for monoubiquitinated Pcna. Nat Commun. (2025) 16:6991. doi: 10.1038/s41467-025-61768-0. PMID: 40739138 PMC12311017

[2] McGehee OC Ebrahim HY Meyer S Ahmed NA Muthumula CMR Dawud D . Safety, pharmacokinetics, translational and molecular mechanistic insights on the prostate cancer recurrence suppressor pseurotin A. Molecules. (2025) 30(19):3963. doi: 10.3390/molecules30193963. PMID: 41097384 PMC12525728

[3] Cruz L Soares P Correia M . Ubiquitin-specific proteases: players in cancer cellular processes. Pharm (Basel). (2021) 14(9):848. doi: 10.3390/ph14090848. PMID: 34577547 PMC8469789

[4] You H Zhang H Jin X Yan Z . Dysregulation of ubiquitination modification in renal cell carcinoma. Front Genet. (2024) 15:1453191. doi: 10.3389/fgene.2024.1453191. PMID: 39748950 PMC11693700

[5] Chen L Liu DH Li YX Yang S Jia WH Peng L . Akebia saponin D targeting ubiquitin carboxyl-terminal hydrolase 4 promotes peroxisome proliferator-activated receptor gamma deubiquitination and activation of brown adipose tissue thermogenesis in obesity. MedComm (2020). (2025) 6:e70420. doi: 10.1002/mco2.70420. PMID: 41104164 PMC12521790

[6] Liu P Guo Y Guo R Yuan T Lai Y Zhu H . Rational design and structure-activity relationship study of novel Josd2 inhibitor against colorectal cancer. Bioorg Chem. (2025) 166:109022. doi: 10.1016/j.bioorg.2025.109022. PMID: 41092805

[7] Hu Y Huang P Jiang F . Identification and analysis of subtypes of liver cancer based on genes related to E3 ubiquitin ligases and deubiquitinating enzymes. Tohoku J Exp Med. (2024) 262:75–84. doi: 10.1620/tjem.2023.j089. PMID: 37880130

[8] Chen X Tian L Zhang L Gao W Yu M Li Z . Deubiquitinase Usp39 promotes Sars-Cov-2 replication by deubiquitinating and stabilizing the envelope protein. Antiviral Res. (2024) 221:105790. doi: 10.1016/j.antiviral.2023.105790. PMID: 38158131

[9] Erven I Abraham E Hermanns T Baumann U Hofmann K . A widely distributed family of eukaryotic and bacterial deubiquitinases related to herpesviral large tegument proteins. Nat Commun. (2022) 13:7643. doi: 10.1038/s41467-022-35244-y. PMID: 36496440 PMC9741609

[10] Li S Song Y Wang K Liu G Dong X Yang F . Usp32 deubiquitinase: cellular functions, regulatory mechanisms, and potential as a cancer therapy target. Cell Death Discov. (2023) 9:338. doi: 10.1038/s41420-023-01629-1. PMID: 37679322 PMC10485055

[11] Hou D Yu D Yang G Hu Y Li H . Research progress in deubiquitinase Otud3. Zhong Nan Da Xue Xue Bao Yi Xue Ban. (2024) 49:1341–52. 10.11817/j.issn.1672-7347.2024.230581 PMC11628234 39788523

[12] Levi-D'Ancona E Stendahl AM Henry-Kanarek BA Davidson RK Walker EM Soleimanpour SA . Mitophagy in the adaptation to pancreatic B cell stress in diabetes. Trends Endocrinol Metab. (2025). doi: 10.1016/j.tem.2025.09.009. PMID: 41109799

[13] Horner SM . Split-site ubiquitination gives Znfx1 new power in Rna defense. Mol Cell. (2025) 85:3734–6. doi: 10.1016/j.molcel.2025.09.022. PMID: 41106363

[14] Zhang Y Du D Fang C Yu X Fang Y Liu X . Epigenetics disruptions enabled by porphyrin-derived metal-organic frameworks disarm resistances to sonocatalytic Ros anti-tumor actions. Fundam Res. (2025) 5:296–306. doi: 10.1016/j.fmre.2022.06.020. PMID: 40166102 PMC11955030

[15] Murali P Kavitha B Narasimhan M . Deubiquitinases and cancer. J Pharm Bioallied Sci. (2024) 16:S4210–20. doi: 10.4103/jpbs.jpbs_517_24. PMID: 40061651 PMC11888656

[16] Wang W Lin H Zheng E Hou Z Liu Y Huang W . Regulation of survivin protein stability by Usp35 is evolutionarily conserved. Biochem Biophys Res Commun. (2021) 574:48–55. doi: 10.1016/j.bbrc.2021.08.050. PMID: 34438346

[17] Dong H Zhang L Chen H Jiang Y Wang P Wang C . Deubiquitinase Zmubp5 is essential for maize kernel development. Plant J. (2025) 124:e70482. doi: 10.1111/tpj.70482. PMID: 41042637

[18] Chen WJ Zhu BL Li WY Shi TS Zhao HY Wang CN . Posttranslational regulation of Creb-binding protein expression by F-box leucine-rich repeat-containing protein 19 and ubiquitin-specific peptidase 14 in the paraventricular nucleus participates in chronic stress-induced symptomatology via the modulation of the hypothalamic–pituitary–adrenal axis. Mol Psychiatry. (2025). doi: 10.1038/s41380-025-03293-6. PMID: 41028568

[19] Li Z Shi C . Ubiquitin-specific protease 34 serves as a novel prognostic biomarker through correlating with immune responses and cell proliferation in acute myeloid leukemia. Discov Oncol. (2025) 16:1914. doi: 10.1007/s12672-025-03662-1. PMID: 41105351 PMC12534648

[20] Frison M Lockey BS Nie Y Golder Z Theiaspra E Ryall CD . Ubiquitin-mediated mitophagy regulates the inheritance of mitochondrial DNA mutations. Science. (2025) 390:156–63. doi: 10.1126/science.adr5438. PMID: 41066576

[21] Zhao Y Zhang Z Xu AM . Heg1 promotes gastric cancer progression by stabilizing Akt1 and is functionally regulated by the deubiquitinase Usp48. Eur J Med Res. (2025) 30:872. doi: 10.1186/s40001-025-02999-1. PMID: 41013721 PMC12465465

[22] Zhou F Li X Sun Y Wang Y Niu K Gao X . Usp39 at the crossroads of cancer immunity: regulating immune evasion and immunotherapy response through Rna splicing and ubiquitin signaling. Front Immunol. (2025) 16:1665775. doi: 10.3389/fimmu.2025.1665775. PMID: 40990019 PMC12450694

[23] Yi J Tavana O Li H Wang D Baer RJ Gu W . Targeting Usp2 regulation of Vprbp-mediated degradation of P53 and Pd-L1 for cancer therapy. Nat Commun. (2023) 14:1941. doi: 10.1038/s41467-023-37617-3. PMID: 37024504 PMC10079682

[24] Qi SM Cheng G Cheng XD Xu Z Xu B Zhang WD . Targeting Usp7-mediated deubiquitination of Mdm2/Mdmx-P53 pathway for cancer therapy: are we there yet? Front Cell Dev Biol. (2020) 8:233. doi: 10.3389/fcell.2020.00233. PMID: 32300595 PMC7142254

[25] Eichhorn PJ Rodón L Gonzàlez-Juncà A Dirac A Gili M Martínez-Sáez E . Usp15 stabilizes Tgf-B receptor I and promotes oncogenesis through the activation of Tgf-B signaling in glioblastoma. Nat Med. (2012) 18:429–35. doi: 10.1038/nm.2619. PMID: 22344298

[26] Zhang L Zhou F Drabsch Y Gao R Snaar-Jagalska BE Mickanin C . Usp4 is regulated by Akt phosphorylation and directly deubiquitylates Tgf-B type I receptor. Nat Cell Biol. (2012) 14:717–26. doi: 10.1038/ncb2522. PMID: 22706160

[27] Dupont S Mamidi A Cordenonsi M Montagner M Zacchigna L Adorno M . Fam/Usp9x, a deubiquitinating enzyme essential for Tgfbeta signaling, controls Smad4 monoubiquitination. Cell. (2009) 136:123–35. doi: 10.1016/j.cell.2008.10.051. PMID: 19135894

[28] Wu Y Yu X Yi X Wu K Dwabe S Atefi M . Aberrant phosphorylation of Smad4 Thr277-mediated Usp9x-Smad4 interaction by free fatty acids promotes breast cancer metastasis. Cancer Res. (2017) 77:1383–94. doi: 10.1158/0008-5472.can-16-2012. PMID: 28115363 PMC5354968

[29] Choi BJ Park SA Lee SY Cha YN Surh YJ . Hypoxia induces epithelial-mesenchymal transition in colorectal cancer cells through ubiquitin-specific protease 47-mediated stabilization of Snail: a potential role of Sox9. Sci Rep. (2017) 7:15918. doi: 10.1038/s41598-017-15139-5. PMID: 29162839 PMC5698333

[30] Lambrus BG Daggubati V Uetake Y Scott PM Clutario KM Sluder G . A Usp28-53bp1-P53-P21 signaling axis arrests growth after centrosome loss or prolonged mitosis. J Cell Biol. (2016) 214:143–53. doi: 10.1083/jcb.201604054. PMID: 27432896 PMC4949452

[31] Yuan J Luo K Zhang L Cheville JC Lou Z . Usp10 regulates P53 localization and stability by deubiquitinating P53. Cell. (2010) 140:384–96. doi: 10.1016/j.cell.2009.12.032. PMID: 20096447 PMC2820153

[32] Pal A Young MA Donato NJ . Emerging potential of therapeutic targeting of ubiquitin-specific proteases in the treatment of cancer. Cancer Res. (2014) 74:4955–66. doi: 10.1158/0008-5472.can-14-1211. PMID: 25172841

[33] Jiang S Tang Y Ma F Niu Y Sun L . Ubiquitin-specific protease 1 facilitates tumor immune escape from natural killer cells and predicts the prognosis in small cell lung cancer. Oncol Res. (2025) 33:213–24. doi: 10.32604/or.2024.046895. PMID: 39735674 PMC11671408

[34] Zhu Y Chen Z Niu K Li M Deng Y Zhang J . Usp33 regulates DNA damage response and carcinogenesis through deubiquitylating and stabilising P53. Cell Prolif. (2025) 58:e13793. doi: 10.1111/cpr.13793. PMID: 39694539 PMC12099211

[35] Park J Shin SC Jin KS Lim MJ Kim Y Kim EE . Usp35 dimer prevents its degradation by E3 ligase Chip through auto-deubiquitinating activity. Cell Mol Life Sci. (2023) 80:112. doi: 10.1007/s00018-023-04740-9. PMID: 37004621 PMC11073304

[36] Wang S Li X Wang T Sun Z Feng E Jin Y . Overexpression of Usp35 enhances the protective effect of Huc-Mscs and their extracellular vesicles in oxygen-glucose deprivation/reperfusion-induced Sh-Sy5y cells via stabilizing Fundc1. Commun Biol. (2024) 7:1330. doi: 10.1038/s42003-024-07024-5. PMID: 39406943 PMC11480199

[37] Zhang J Chen Y Chen X Zhang W Zhao L Weng L . Deubiquitinase Usp35 restrains Sting-mediated interferon signaling in ovarian cancer. Cell Death Differ. (2021) 28:139–55. doi: 10.1038/s41418-020-0588-y. PMID: 32678307 PMC7853139

[38] Dai DL Xie C Zhong LY Liu SX Zhang LL Zhang H . Axin1 boosts antiviral response through Irf3 stabilization and induced phase separation. Signal Transduct Target Ther. (2024) 9:281. doi: 10.1038/s41392-024-01978-y. PMID: 39384753 PMC11464762

[39] Cao J Wu T Zhou T Jiang Z Ren Y Yu J . Usp35 promotes the growth of Er positive breast cancer by inhibiting ferroptosis via Brd4-Slc7a11 axis. Commun Biol. (2025) 8:64. doi: 10.1038/s42003-025-07513-1. PMID: 39820080 PMC11739500

[40] Guo Y Lin Z Zhou Z Zhang W Mao S Shan Z . Oncogenic and immunological functions of Usp35 in pan-cancer and its potential as a biomarker in kidney clear cell carcinoma. BMC Cancer. (2025) 25:617. doi: 10.1186/s12885-025-13964-w. PMID: 40188027 PMC11972461

[41] Tsai IC Adams KA Tzeng JA Shennib O Tan PL Katsanis N . Genome-wide suppressor screen identifies Usp35/Usp38 as therapeutic candidates for ciliopathies. JCI Insight. (2019) 4(22):e130516. doi: 10.1172/jci.insight.130516. PMID: 31723061 PMC6948861

[42] Liu C Wang L Chen W Zhao S Yin C Lin Y . Usp35 activated by Mir let-7a inhibits cell proliferation and Nf-Kb activation through stabilization of Abin-2. Oncotarget. (2015) 6:27891–906. doi: 10.18632/oncotarget.4451. PMID: 26348204 PMC4695033

[43] Zhang H Zhu J He R Xu L Chen Y Yu H . Deubiquitination enzyme Usp35 negatively regulates Mavs signaling to inhibit anti-tumor immunity. Cell Death Dis. (2025) 16:138. doi: 10.1038/s41419-025-07411-8. PMID: 40016186 PMC11868397

[44] Zhu S Zhang X Liu W Zhou Z Xiong S Li J . Ubiquitination in cancer: mechanisms and therapeutic opportunities. Cancer Commun (Lond). (2025) 45:1128–61. doi: 10.1002/cac2.70044. PMID: 40537889 PMC12479132

[45] Altman DG McShane LM Sauerbrei W Taube SE . Reporting recommendations for tumor marker prognostic studies (Remark): explanation and elaboration. PloS Med. (2012) 9:e1001216. doi: 10.1371/journal.pmed.1001216. PMID: 22675273 PMC3362085

[46] Mei Y Hahn AA Hu S Yang X . The Usp19 deubiquitinase regulates the stability of C-Iap1 and C-Iap2. J Biol Chem. (2011) 286:35380–7. doi: 10.1074/jbc.m111.282020. PMID: 21849505 PMC3195621

[47] Koschel J Nishanth G Just S Harit K Kröger A Deckert M . Otub1 prevents lethal hepatocyte necroptosis through stabilization of C-Iap1 during murine liver inflammation. Cell Death Differ. (2021) 28:2257–75. doi: 10.1038/s41418-021-00752-9. PMID: 33712742 PMC8257688

[48] Wang S Wang T Zhang X Cheng S Chen C Yang G . The deubiquitylating enzyme Usp35 restricts regulated cell death to promote survival of renal clear cell carcinoma. Cell Death Differ. (2023) 30:1757–70. doi: 10.1038/s41418-023-01176-3. PMID: 37173391 PMC10307860

[49] Pan Q Zhong S Wang H Wang X Li N Li Y . The Zmynd8-regulated mevalonate pathway endows Yap-high intestinal cancer with metabolic vulnerability. Mol Cell. (2021) 81:2736–2751.e8. doi: 10.1016/j.molcel.2021.04.009. PMID: 33932349

[50] Lin G Huang T Zhang X Wang G . Deubiquitinase Usp35 stabilizes Brpf1 to activate mevalonate (Mva) metabolism during prostate tumorigenesis. Cell Death Discov. (2022) 8:453. doi: 10.1038/s41420-022-01231-x. PMID: 36357379 PMC9649703

[51] Shimano H Sato R . Srebp-regulated lipid metabolism: convergent physiology - divergent pathophysiology. Nat Rev Endocrinol. (2017) 13:710–30. doi: 10.1038/nrendo.2017.91. PMID: 28849786

[52] Cruz-Gregorio A Aranda-Rivera AK Pedraza-Chaverri J . Nuclear factor erythroid 2-related factor 2 in human papillomavirus-related cancers. Rev Med Virol. (2022) 32:e2308. doi: 10.1002/rmv.2308. PMID: 34694662

[53] Zhang D Li J Zhang C Xue J Li P Shang K . The deubiquitinating enzyme Usp35 regulates the stability of Nrf2 protein. Open Life Sci. (2024) 19:20220935. doi: 10.1515/biol-2022-0935. PMID: 39156988 PMC11330172

[54] Inoue Y Itoh Y Sato K Kawasaki F Sumita C Tanaka T . Regulation of epithelial-mesenchymal transition by E3 ubiquitin ligases and deubiquitinase in cancer. Curr Cancer Drug Targets. (2016) 16:110–8. doi: 10.2174/1568009616666151112122126. PMID: 26560121

[55] Ma C Tian Z Wang D Gao W Qian L Zang Y . Ubiquitin-specific protease 35 promotes gastric cancer metastasis by increasing the stability of Snail1. Int J Biol Sci. (2024) 20:953–67. doi: 10.7150/ijbs.87176. PMID: 38250150 PMC10797686

[56] Lv T Zhang B Jiang C Zeng Q Yang J Zhou Y . Usp35 promotes hepatocellular carcinoma progression by protecting Pkm2 from ubiquitination-mediated degradation. Int J Oncol. (2023) 63(3):953–967. doi: 10.3892/ijo.2023.5561. PMID: 37594129 PMC10552738

[57] Chen D Wang Y Lu R Jiang X Chen X Meng N . E3 ligase Zfp91 inhibits hepatocellular carcinoma metabolism reprogramming by regulating Pkm splicing. Theranostics. (2020) 10:8558–72. doi: 10.7150/thno.44873. PMID: 32754263 PMC7392027

[58] Chen G Shi Y Zhang S Zhang X Li G Jiang C . Usp35 promotes hepatocellular carcinoma proliferation through Gasc1-mediated Rock2 upregulation. Transl Oncol. (2025) 58:102430. doi: 10.1016/j.tranon.2025.102430. PMID: 40424935 PMC12159206

[59] Cheng TC Tu SH Chen LC Chen MY Chen WY Lin YK . Down-regulation of A-L-fucosidase 1 expression confers inferior survival for triple-negative breast cancer patients by modulating the glycosylation status of the tumor cell surface. Oncotarget. (2015) 6:21283–300. doi: 10.18632/oncotarget.4238. PMID: 26204487 PMC4673265

[60] Baudot AD Crighton D O'Prey J Somers J Sierra Gonzalez P Ryan KM . P53 directly regulates the glycosidase Fuca1 to promote chemotherapy-induced cell death. Cell Cycle. (2016) 15:2299–308. doi: 10.1080/15384101.2016.1191714. PMID: 27315169 PMC5004703

[61] Xiao Y Jiang X Yin K Miao T Lu H Wang W . Usp35 promotes cell proliferation and chemotherapeutic resistance through stabilizing Fuca1 in colorectal cancer. Oncogenesis. (2023) 12:12. doi: 10.1038/s41389-023-00458-2. PMID: 36864055 PMC9981583

[62] Liu T Jiang L Tavana O Gu W . The deubiquitylase Otub1 mediates ferroptosis via stabilization of Slc7a11. Cancer Res. (2019) 79:1913–24. doi: 10.1158/0008-5472.can-18-3037. PMID: 30709928 PMC6467774

[63] Cao J Wu D Wu G Wang Y Ren T Wang Y . Usp35, regulated by estrogen and Akt, promotes breast tumorigenesis by stabilizing and enhancing transcriptional activity of estrogen receptor A. Cell Death Dis. (2021) 12:619. doi: 10.1038/s41419-021-03904-4. PMID: 34131114 PMC8206120

[64] Lian W Hong C Chen D Wang C . Usp35 promotes breast cancer progression by regulating Pfk-1 ubiquitination to mediate glycolysis. Am J Physiol Cell Physiol. (2025) 328:C355–66. doi: 10.1152/ajpcell.00733.2024. PMID: 39714773

[65] Shu HB Wang YY . Adding to the sting. Immunity. (2014) 41:871–3. doi: 10.1016/j.immuni.2014.12.002. PMID: 25526298

[66] Ethiraj P Veerappan K Samuel S Sivapatham S . Interferon B improves the efficacy of low dose cisplatin by inhibiting Nf-Kb/P-Akt signaling on Hela cells. BioMed Pharmacother. (2016) 82:124–32. doi: 10.1016/j.biopha.2016.04.058. PMID: 27470347

[67] Grabosch S Bulatovic M Zeng F Ma T Zhang L Ross M . Cisplatin-induced immune modulation in ovarian cancer mouse models with distinct inflammation profiles. Oncogene. (2019) 38:2380–93. doi: 10.1038/s41388-018-0581-9. PMID: 30518877 PMC6440870

[68] Li W Li J Li W Li J Zheng W Cheng J . Fus-stabilized Usp35 promotes growth, invasion and angiogenesis in Nsclc through deubiquitinating Vegfa. Gen Physiol Biophys. (2024) 43:301–12. doi: 10.4149/gpb_2024010. PMID: 38953570

[69] Liu C Chen Z Ding X Qiao Y Li B . Ubiquitin-specific protease 35 (Usp35) mediates cisplatin-induced apoptosis by stabilizing Birc3 in non-small cell lung cancer. Lab Invest. (2022) 102:524–33. doi: 10.1038/s41374-021-00725-z. PMID: 35022505

[70] Lin CY Hung CC Wang CCN Lin HY Huang SH Sheu MJ . Demethoxycurcumin sensitizes the response of non-small cell lung cancer to cisplatin through downregulation of Tp and Ercc1-related pathways. Phytomedicine. (2019) 53:28–36. doi: 10.1016/j.phymed.2018.08.005. PMID: 30668408

[71] Wang W Wang M Xiao Y Wang Y Ma L Guo L . Usp35 mitigates endoplasmic reticulum stress-induced apoptosis by stabilizing Rrbp1 in non-small cell lung cancer. Mol Oncol. (2022) 16:1572–90. doi: 10.1002/1878-0261.13112. PMID: 34618999 PMC8978513

[72] Pan Y Cao F Guo A Chang W Chen X Ma W . Endoplasmic reticulum ribosome-binding protein 1, Rrbp1, promotes progression of colorectal cancer and predicts an unfavourable prognosis. Br J Cancer. (2015) 113:763–72. doi: 10.1038/bjc.2015.260. PMID: 26196185 PMC4559827

[73] Tang Z Jiang W Mao M Zhao J Chen J Cheng N . Deubiquitinase Usp35 modulates ferroptosis in lung cancer via targeting ferroportin. Clin Transl Med. (2021) 11:e390. doi: 10.1002/ctm2.390. PMID: 33931967 PMC8087931

[74] Wang K An L Zang A Huo Y . Chidamide impedes glycolysis but increases ferroptosis and cisplatin sensitivity of lung cancer cells through downregulating Usp35. BMC Cancer. (2025) 25:1504. doi: 10.1186/s12885-025-14925-z. PMID: 41044693 PMC12495749

[75] Schmidt A Schwerd T Hamm W Hellmuth JC Cui S Wenzel M . 5'-Triphosphate Rna requires base-paired structures to activate antiviral signaling via Rig-I. Proc Natl Acad Sci USA. (2009) 106:12067–72. doi: 10.1073/pnas.0900971106. PMID: 19574455 PMC2705279

[76] Blaszczyk K Nowicka H Kostyrko K Antonczyk A Wesoly J Bluyssen HA . The unique role of Stat2 in constitutive and Ifn-induced transcription and antiviral responses. Cytokine Growth Factor Rev. (2016) 29:71–81. doi: 10.1016/j.cytogfr.2016.02.010. PMID: 27053489

[77] Jain P Wang ML . Mantle cell lymphoma in 2022-a comprehensive update on molecular pathogenesis, risk stratification, clinical approach, and current and novel treatments. Am J Hematol. (2022) 97:638–56. doi: 10.1002/ajh.26523. PMID: 35266562

[78] Harmanen M Hujo M Sund R Sorigue M Khan M Prusila R . Survival of patients with mantle cell lymphoma in the rituximab era: retrospective binational analysis between 2000 and 2020. Br J Haematol. (2023) 201:64–74. doi: 10.1111/bjh.18597. PMID: 36513500

[79] Zou Z Chen S Wu Y Ji S . The Usp35-Cxcr3 axis plays an oncogenic role in Jeko-1 mantle cell lymphoma cells. Integr Biol (Camb). (2024) 16(1):64–74. doi: 10.1093/intbio/zyae021. PMID: 39591978

[80] Park J Kwon MS Kim EE Lee H Song EJ . Usp35 regulates mitotic progression by modulating the stability of Aurora B. Nat Commun. (2018) 9:688. doi: 10.1038/s41467-018-03107-0. PMID: 29449677 PMC5814453

[81] Harrigan JA Jacq X Martin NM Jackson SP . Deubiquitylating enzymes and drug discovery: emerging opportunities. Nat Rev Drug Discov. (2018) 17:57–78. doi: 10.1038/nrd.2017.152. PMID: 28959952 PMC7097658

[82] Zhang Y Yao K Yu Y Ni S Zhang L Wang W . Effects of 1.8 Ghz radiofrequency radiation on protein expression in human lens epithelial cells. Hum Exp Toxicol. (2013) 32:797–806. doi: 10.1177/0960327112472353. PMID: 23338683

[83] Wang Y Yao X Lu Y Ruan J Yang Z Wang C . A Protac-based cuproptosis sensitizer in lung cancer therapy. Adv Mater. (2025) 37:e2501435. doi: 10.1002/adma.202501435. PMID: 40495637

[84] Wang C Zhang Y Chen W Wu Y Xing D . New-generation advanced Protacs as potential therapeutic agents in cancer therapy. Mol Cancer. (2024) 23:110. doi: 10.1186/s12943-024-02024-9. PMID: 38773495 PMC11107062

[85] Oon CE Anbazhagan P Tan CT . Therapeutic potential of targeting ubiquitin-specific proteases in colorectal cancer. Drug Discov Today. (2025) 30:104356. doi: 10.1016/j.drudis.2025.104356. PMID: 40216291

[86] Murgai A Sosič I Gobec M Lemnitzer P Proj M Wittenburg S . Targeting the deubiquitinase Usp7 for degradation with Protacs. Chem Commun (Camb). (2022) 58:8858–61. doi: 10.1039/d2cc02094g. PMID: 35852517 PMC9710854

[87] Zhang J Chen C Chen X Liao K Li F Song X . Linker-free Protacs efficiently induce the degradation of oncoproteins. Nat Commun. (2025) 16:4794. doi: 10.1038/s41467-025-60107-7. PMID: 40410168 PMC12102262

[88] Li R Zhang S Xu Y Liu R Dai Z . Nanoenabled strategies enhancing Protacs for cancer therapy. Bioconjug Chem. (2025) 36:1582–7. doi: 10.1021/acs.bioconjchem.5c00305. PMID: 40726077

[89] Arenas-Moreira M Ocaña A Bravo I Alonso-Moreno C . Protac delivery systems: innovative approaches for cancer treatment. BioMed Pharmacother. (2026) 194:118892. doi: 10.1016/j.biopha.2025.118892. PMID: 41389627

[90] Sheng J Ma T Wu Y Wang M . Programmable Protac delivery for precise and spatiotemporal protein degradation. Chem Commun (Camb). (2026) 62:2414–27. doi: 10.1039/d5cc05651a. PMID: 41489095

[91] Chan A Haley RM Najar MA Gonzalez-Martinez D Bugaj LJ Burslem GM . Lipid-mediated intracellular delivery of recombinant Bioprotacs for the rapid degradation of undruggable proteins. Nat Commun. (2024) 15:5808. doi: 10.1038/s41467-024-50235-x. PMID: 38987546 PMC11237011

[92] De Leon G Zhang L Siddiqui NA Naguib N Chen F Padmanabhan R . An ultrasmall core-shell silica nanoparticle improves antitumour immunity and survival by remodelling suppressive melanoma microenvironments. Nat Nanotechnol. (2026) 21:311–22. doi: 10.1038/s41565-025-02083-z. PMID: 41461940 PMC12916478

[93] Zhao X Liu D Li G Xu W Wu G . Nanomedicines reshape the tumor microenvironment: multidimensional strategies from modulating "barriers" to metabolic intervention. Int J Nanomed. (2026) 21:570411. doi: 10.2147/ijn.s570411. PMID: 41858588 PMC12998913

[94] Paulus A Akhtar S Caulfield TR Samuel K Yousaf H Bashir Y . Coinhibition of the deubiquitinating enzymes, Usp14 and Uchl5, with Vlx1570 is lethal to Ibrutinib- or Bortezomib-resistant Waldenstrom macroglobulinemia tumor cells. Blood Cancer J. (2016) 6:e492. doi: 10.1038/bcj.2016.93. PMID: 27813535 PMC5148058

[95] Zhang Q Liu YJ Li JP Zeng SH Shen H Han M . Usp35 is a potential immunosuppressive factor in skin cutaneous melanoma. J Inflammation Res. (2022) 15:3065–82. doi: 10.2147/jir.s362619. PMID: 35637872 PMC9148213

[96] Chen W Wang L Fan H Li L Zhang L Li R . Usp35 acts as a deubiquitinating enzyme for Id3 to promote immune escape in colorectal cancer. Adv Sci (Weinh). (2026) 13:e16588. doi: 10.1002/advs.202516588. PMID: 41486422 PMC13042420

